# Case report: A lung squamous cell carcinoma patient with a rare *EGFR* G719X mutation and high *PD-L1* expression showed a good response to anti-*PD1* therapy

**DOI:** 10.3389/fonc.2024.1283008

**Published:** 2024-01-31

**Authors:** Zhen-feng Zhu, Xu-xia Bao, Hong-yan Shi, Xi-xi Gu

**Affiliations:** ^1^ Department of Integrative Medicine, Zhongshan Hospital, Fudan University, Shanghai, China; ^2^ Department of Integrative Medicine, Shanghai Medical College, Fudan University, Shanghai, China; ^3^ Medical Department, Genecast Biotechnology Co., Ltd, Wuxi, China

**Keywords:** lung squamous cell carcinoma, EGFR G719X mutation, anti-PD1 therapy, afatinib, inflammatory microenvironment

## Abstract

Lung cancer treatment has transitioned fully into the era of immunotherapy, yielding substantial improvements in survival rate for patients with advanced non-small cell lung cancer (NSCLC). In this report, we present a case featuring a rare epidermal growth factor receptor (*EGFR*) mutation accompanied by high programmed death-ligand 1 (*PD-L1*) expression, demonstrating remarkable therapeutic efficacy through a combination of immunotherapy and chemotherapy. A 77-year-old male with no family history of cancer suffered from upper abdominal pain for more than half months in August 2020 and was diagnosed with stage IV (cT3N3M1c) lung squamous cell carcinoma (LUSC) harboring both a rare *EGFR* p.G719C mutation and high expression of *PD-L1* (tumor proportion score [TPS] = 90%). Treatment with the second-generation targeted therapy drug Afatinib was initiated on September 25, 2020. However, resistance ensued after 1.5 months of treatment. On November 17, 2020, immunotherapy was combined with chemotherapy (Sintilimab + Albumin-bound paclitaxel + Cisplatin), and a CT scan conducted three months later revealed significant tumor regression with a favorable therapeutic effect. Subsequently, the patient received one year of maintenance therapy with Sintilimab, with follow-up CT scans demonstrating subtle tumor shrinkage (stable disease). This case provides evidence for the feasibility and efficacy of immunotherapy combined with chemotherapy in the treatment of *EGFR*-mutated and *PD-L1* highly expressed LUSC.

## 1 Introduction

Lung cancer ranks as a foremost contributor to cancer-related mortality, standing among the most prevalent and lethal malignancies worldwide (1). Non-small cell lung cancer (NSCLC) accounts for more than 85% of all lung cancer cases, with lung adenocarcinoma (LUAD) and lung squamous cell carcinoma (LUSC) representing predominant subtypes. Specifically, LUSC comprises approximately 20-30% of NSCLC cases (2). The epidermal growth factor receptor (*EGFR*) is a transmembrane receptor tyrosine kinase that regulates cell growth and differentiation in normal cells (3). However, mutations in *EGFR* lead to its sustained activation, consequently promoting abnormal proliferation and survival of tumor cells (4). *EGFR* mutations predominantly manifest in LUAD, occurring in approximately 27% of cases. The common *EGFR* mutations include exon 19 deletions (comprising approximately 30-45% of *EGFR* mutations) and exon 21 L858R point mutations (comprising approximately 40-45% of *EGFR* mutations). Additionally, other *EGFR* mutations such as G719X (G719A, G719C, G719S), S768I, and L861Q are considered rare *EGFR* mutations, accounting for a lower proportion (5). In contrast, *EGFR* mutations in LUSC are relatively infrequent, accounting for merely about 7% or less of cases (6). Among the *EGFR* mutations detected in LUSC, reports on the specific mutation subtype *EGFR* G719C are extremely limited.

With the advancement of molecular biology in lung cancer, immunotherapy and targeted therapy have become the standard treatment approaches for advanced lung cancer (7). Studies have revealed significant survival benefits for stage II to IIIA NSCLC patients carrying common *EGFR* mutations (Ex19del or L858R) after complete resection (5-year survival rate of 5%). Additionally, NSCLC patients with stage II to IIIA *EGFR* mutation-positive tumors who received immunotherapy showed significantly higher median progression-free survival (PFS) compared to those who received chemotherapy alone (30.8 months vs. 19.8 months), along with a higher 3-year PFSrate (39.6% vs. 32.5%) (8). In a rare case of stage IB LUSC, the patient exhibited both *EGFR* p.L858R mutation and high programmed death-ligand 1 (*PD-L1*) expression. After two cycles of immunotherapy, the tumor significantly regressed, and subsequent right upper thoracoscopic lobectomy with mediastinal lymph node dissection led to complete pathological remission (9). The current consensus is that NSCLC patients with uncommon *EGFR* mutations typically exhibit resistance to *EGFR* tyrosine kinase inhibitors (*EGFR*-TKIs), resulting in overall poor benefits from first-generation *EGFR*-TKIs (5). However, studies have reported potential benefits from the second-generation *EGFR*-TKI drug, Afatinib, for patients carrying *EGFR* G719X mutations (10). A comprehensive meta-analysis of Afatinib revealed that patients with *EGFR* exon 20 insertions responded poorly to the drug, while those with G719X, S768I, and L861Q mutations responded well, with PFS outcomes comparable to common *EGFR* mutations (11). Another study found that a patient with G719D and 21 (L861Q) co-mutations in a poorly differentiated stage IVB NSCLC had a survival period of 13 months and was currently undergoing treatment with Amolertinib (12). Nevertheless, the simultaneous presence of *EGFR* driver gene mutations and *PD-L1* overexpression in LUSC is extremely rare, and patients with *PD-L1* negativity (<1%) often have longer median overall survival (OS) than *PD-L1*-positive patients (15.61 vs. 7.40 months, P=0.0138) (13, 14). Clinical trials have demonstrated the safety and efficacy of programmed cell death-1 (PD-1)/*PD-L1* inhibitors in resectable NSCLC neoadjuvant therapy (15). Afatinib exhibits heterogeneity across different mutation genotypes, with *TP53* mutation patients receiving less survival benefit than *TP53* wild-type patients (16). Hence, further research into novel drugs and combination treatment strategies is crucial for expanding the clinical benefit population and improving the prognosis of patients with *EGFR* G719C uncommon mutation combined with high *PD-L1* expression.

This study reports a case of LUSC in which the patient simultaneously exhibited *EGFR* G719X mutation and high *PD-L1* expression, and received treatment with Afatinib. However, after one and a halfmonths of treatment, the patient developed drug resistance. Therefore, exploring potential resistance mechanisms is a challenging clinical issue that needs to be addressed. Through this research, we aim to provide insights into the treatment of LUSC patients with the rare *EGFR* G719X mutation and concurrent high *PD-L1* expression.

## 2 Case presentation

**Figure 1:**
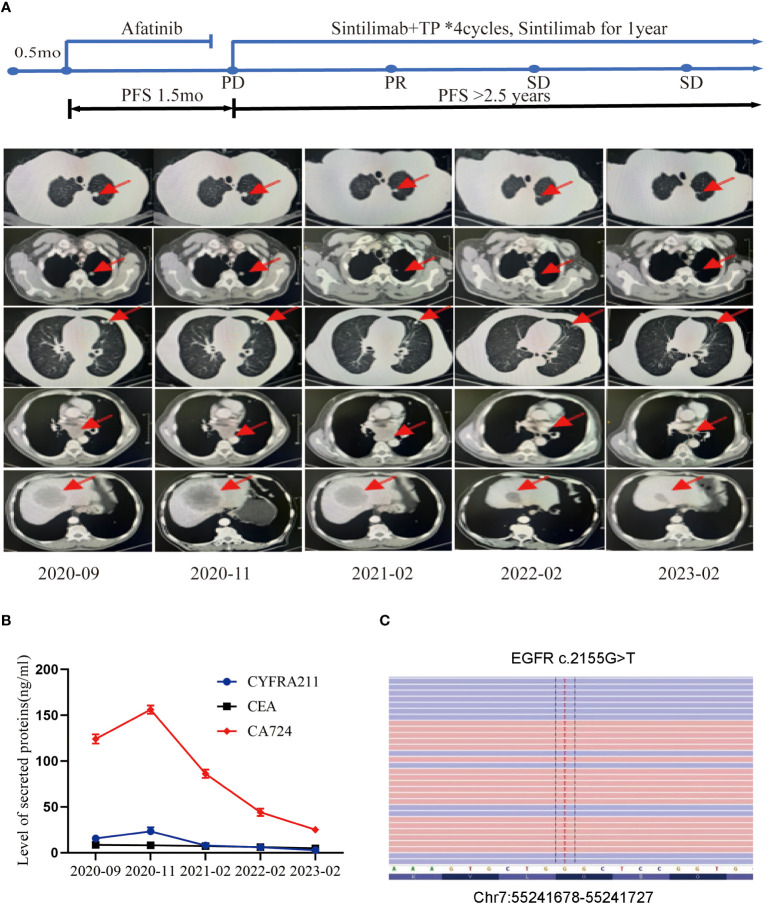
Tumor progression of the patient during the treatments. **(A)** The timeline of the therapies and imaging results of the computed tomography (CT) can during diagnosis and treatment at different time points. **(B)** The levels of secreted protein during treatment at different time points. **(C)** The site of EGFR mutation.

**Figure 2:**
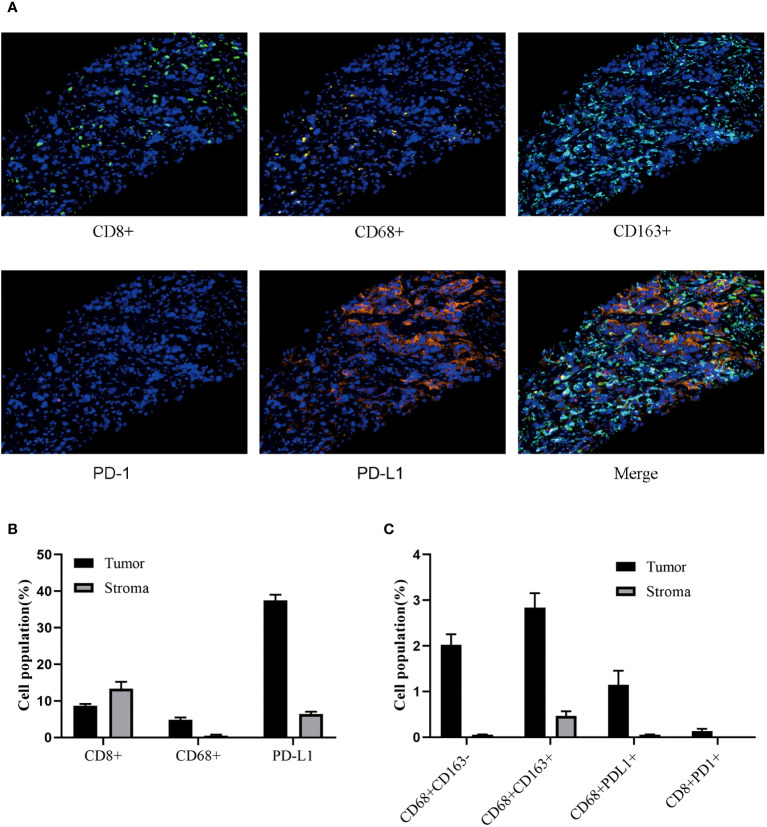
The Immune microenvironment status of liver metastases. **(A)** Multicolor analysis is used to detect the phenotypes of different cell populations. **(B, C)** Perform cluster analysis on various cell types in the stroma and tumor regions.

A 77-year-old male with no family history of cancer suffered from upper abdominal pain for more than half a month in August 2020. An enhanced CT examination revealed suspicious malignant tumors in the left lung, left pleura, multiple lymph nodes (mediastinal, bilateral hilar, left clavicular area, hepatoduodenal ligament area, and retroperitoneum), as well as at the junction of the left and right lobes of the liver near the diaphragm apex and the right adrenal gland (**Figure 1A**). Additionally, the level of the serum tumor biomarkers *CYFRA211*, *CEA*, and *CA724* were elevated above normal levels (**Figure 1B**). A liver biopsy performed on September 22, 2020, showed significant heterogeneity in tumor cells, indicating possible metastatic squamous cell carcinoma. Genetic testing of the liver metastasis tissue revealed *EGFR* p.G719C (mutation frequency 10.81%, number of supported reads 138; **Figure 1C**) and *PIK3CA* mutation. Immunohistochemical staining of the liver metastasis lesion showed high infiltration of *CD8* ^+^ and *PD-L1* in both the tumor area and stroma, with *CD68* ^+^ macrophages including M1-type macrophages (*CD68* ^+^ *CD163* ^-^) and M2-type macrophages (*CD68* ^+^ *CD163* ^+^) having good infiltration in the tumor area. Additionally, a minimal number of exhausted T cells (*CD8* ^+^ *PD1* ^+^) were observed in the tumor (**Figures 2A–C**). Based on the above data, the patient was diagnosed with the rare *EGFR*-mutated LUSC at stage cT3N3M1c with high PD-L1 expression (TPS [tumor proportion score] = 90%) and high tumor mutational burden (TMB, 13.63 mutations/mb). According to the National Comprehensive Cancer Network (NCCN) guidelines, the second-generation *EGFR*-TKI was administered as a once-daily oral dose of 40 mg on September 25, 2020.

However, the CT scan conducted in November 2020 revealed an enlargement of the lung and liver lesions in comparison to the previous scan, leading to disease progression (PD) as per the Response Evaluation Criteria in Solid Tumors (RECIST) scoring criteria (**Figure 1A**). Additionally, the levels of tumor markers *CYFRA211*, *CEA*, and *CA724* experienced further elevation (**Figure 1B**).

Upon developing resistance to Afatinib, a second mediastinal lymph node biopsy was performed, which revealed lung metastatic squamous cell carcinoma. We compared the results of genetic testing of liver metastatic tissue at the time of diagnosis and mediastinal lymph node metastatic tissue at the time of progression on afatinib therapy (**Table 1**). NGS sequencing of the mediastinal lymph node tissue identified three pathogenic/likely pathogenic mutations: *EGFR* p.G719C (14.62%), *PIK3CA* p.E545K (12.16%), and *TP53* p.Q331* (23.08%). Immunogenomic analysis indicated a high tumor mutation burden (17.52/Mb) and 99% expression of *PD-L1* in tumor cells. Given the patient’s age, physical condition, and in accordance with the NCCN guidelines, a combination of immunotherapy and chemotherapy was administered on November 17, 2020, for a total of four cycles. The treatment regimen included albumin-bound paclitaxel (200mg/m2, d1) + cisplatin (75mg/m2, d1) in combination with sintilimab (200mg, d0), with each cycle repeated every three weeks.

**Table 1:** Comparison of genetic testing results between liver metastasis tissue at diagnosis and mediastinal lymph node metastasis tissue during afatinib treatment.

Gene	Exon	Transcript Chromosome Mutation initiation site	Nucleotide changes Amino acid changes	Liver tissue	Mediastinal lymph node tissue
ARID1A	20	NM_006015.6 chr1 27106915	c.6526C>G p.Q2176E	13.5%	9.92%
CDKN2C	3	NM_001262.3 chr1 51439661	c.226G>T p.D76Y	12.33%	17.15%
EGFR	18	NM_005228.5 chr7 55241678	c.2126A>C p.E709A	12.61%	15.53%
EGFR	18	NM_005228.5 chr7 55241707	c.2155G>T p.G719C	–	14.64%
ETV6	4	NM_001987 chr12 12006401	c.369G>C p.Q123H	–	8.94%
FUBP1	2	NM_003902.5 chr1 78435672	c.148G>C p.G50R	14.36%	14.34%
GRIN2A	14	NM_000833.5 chr16 9858167	c.3234G>C p.K1078N	13.04%	14.56%
INPP4A	12	NM_001134224 chr2 99162511	c.1029G>A p.M343I	–	11.75%
IRS2	1	NM_003749 chr13 110436155	c.2246C>T p.S749F	–	13.56%
JAK2	3	5022144	c.157G>C p.D53H	–	8.97%
MDM2	4	NM_001367990.1 chr12 69210640	c.205G>C p.E69Q	12.65%	9.18%
MTOR	6	NM_004958.4 chr1 11314012	c.724G>C p.E242Q	13.07%	8.88%
NF2	14	NM_000268.4 chr22 30074302	c.1564G>T p.E522*	23.78%	19.31%
PIK3CA	10	NM_006218.4 chr3 178936091	c.1633G>A p.E545K	22.89%	12.16%
PLCG2	30	NM_002661 chr16 81973519	c.3336C>G p.I1112M	–	13.67%
RAD21	9	NM_006265.3 chr8 117866648	c.997G>C p.D333H	12.94%	12.79%
RASA1	19	NM_002890.3 chr5 86675622	c.2558C>G p.S853*	16.87%	18.31%
TERT	–	NM:198253 chr5 1295228	. C228T	32.7%	–
TET2	11	NM_001127208.3 chr4 106197128	c.5461G>A p.D1821N	16.11%	20.34%
TP53	9	NM_000546.6 chr17 7576855	c.991C>T p.Q331*	13.55%	23.08%
TSHR	10	NM_000369.5 chr14 81610629	c.2227G>T p.E743*	11.31%	17.12%

The symbol * represents translation termination of messenger RNA.

By February 2021, the CT examination showed significant reduction in the size of the lesions in the left lung, liver, and adrenal gland, accompanied by a decrease in size in the mediastinal lymph nodes compared to the previous examination (**Figure 1A**). As per the RECIST scoring criteria, the assessment result indicated a partial response (PR). The levels of tumor marker levels also significantly decreased (**Figure 1B**).

**Figure 3:**
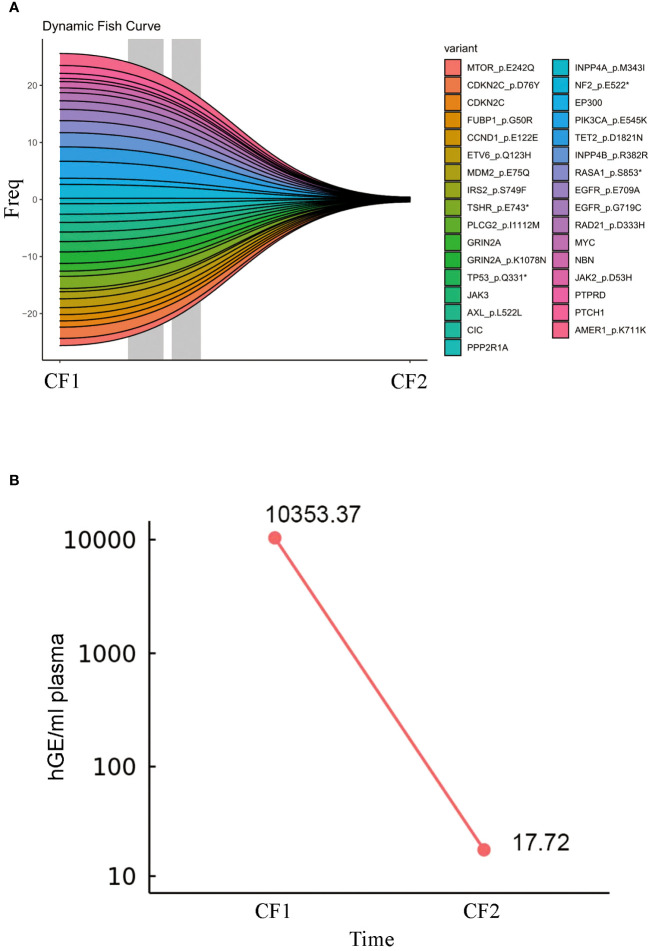
Circulating tumor DNA change during ICI treatments. **(A)** The fish plot illustrates the frequency and types of gene mutations. **(B)** The level of ctDNA.CF1: 2020.11, CF2:2022.9. The symbol * represents translation termination of messenger RNA.

The patient continued to receive maintenance therapy with sintilimab for one year. In February 2022, a follow-up CT scan indicated tumor stabilization (**Figures 1A, B**). Blood plasma circulating tumor DNA (ctDNA)-NGS tests were performed simultaneously in November 2020 and September 2022, revealing a substantial decrease in the number and frequency of gene mutations (Fishplot) (**Figure 3A**). Moreover, quantitative analysis of ctDNA levels demonstrated a significant reduction (17.72 vs. 10353.37 HGE/ml), consistent with the radiological evaluation results (**Figure 3B**).

## 3 Discussion

In LUAD, the most common mutated genes are *KRAS* and *EGFR*, while in LUSC, common mutated genes include *TP53* and *CDKN2A*. The *EGFR* G719C mutation is extremely rare in LUSC (6). The frequency of *EGFR* genomic alterations (including mutations and amplifications) and *ALK* rearrangements in the Chinese NSCLC population is significantly higher than in Western populations, at 50.1% and 7.8%, respectively. Among them, approximately 7.4% of patients carry concurrent activating and uncommon mutations, while 11.6% of patients carry only uncommon *EGFR* mutations, with uncommon *EGFR* mutations often co-occurring with genomic alterations in *ALK, CDKN2A, NTRK3, TSC2*, and *KRAS* (17). Recent research reported a case of LUSC with an *EGFR* G719X mutation. The patient underwent *EGFR*-TKI treatment and achieved an overall survival duration of 25.4 months (18). Mutations in *KRAS* and *EGFR* are usually mutually exclusive, but when coexistent, *KRAS* mutations may lead to resistance to *EGFR* inhibitors (19). Among 101 patients with *EGFR* mutations, strong positive *PD-L1* expression significantly reduced objective response rate and PFS compared to weak positive or negative *PD-L1* expression. Moreover, higher *PD-L1* expression was observed in primary resistance patients to *EGFR*-TKIs (20). Based on the CT results and tumor biomarkers, this study indicated the development of resistance to Afatinib as early as September 2020, consistent with previous findings suggesting that positive *PD-L1* expression mainly occurs in patients with primary resistance to *EGFR*-TKIs. Another study found that patients with *PD-L1* ≥1% exhibited a higher rate of primary resistance to *EGFR*-TKI treatment. Regarding PFS, the median for patients with *PD-L1*≥1%, *PD-L1*≥25%, and *PD-L1*≥50% were 2.1 months, 1.8 months, and 1.6 months, respectively (21). These results suggest that high *PD-L1* expression may be a contributing factor to TKI resistance and rapid progression in LUSC.

Furthermore, we detected mutations in *PIK3CA* p.E545K and *TP53* p.Q331*. *TP53* mutations may lead to impaired immune recognition and antigen presentation of tumor cells, making it difficult for the immune system to recognize and attack tumor cells (22). *PIK3CA* mutations may interfere with the function and metabolism of immune cells, disrupting the tumor immune surveillance mechanism (23). This state of immune evasion may contribute to the development of resistance to *EGFR*-TKIs in tumor cells. Additionally, *TP53* and *PIK3CA* mutations may interact with other oncogenic signaling pathways, such as RAS/MAPK and PI3K/AKT, further enhancing the survival and proliferation capabilities of tumor cells, thereby reducing the effectiveness of *EGFR*-TKIs (24).

PD-1 is a protein localized on the surface of immune cells, while *PD-L1* is a protein expressed on the surface of cancer cells. Immune checkpoint inhibitors (ICI) can block the binding between PD-1 and *PD-L1*, thereby restoring the immune system’s ability to attack cancer cells (25). In this study, while the high PD-L1 expression suggested a favorable response to monotherapy, the decision to combine immunotherapy with chemotherapy was guided by several factors including the patient’s age, overall physical condition, and the desire for a comprehensive and aggressive treatment approach. The rationale behind the combination therapy was to optimize treatment efficacy while minimizing potential risks and maximizing the chances of a robust response given the advanced stage of the disease. The patient was old, though in good health. More importantly, this patient had a large tumor load with liver metastases and mediastinal lymphoma metastasis, and combination chemotherapy and immunotherapy was chosen to reduce the tumor load more rapidly. On the other hand, the patient combined multiple tumor drivers, including high PDL1 expression combined with G719X mutation and also squamous carcinoma, and there may be the influence of factors other than immunity, so the combination therapy was used in order to comprehensively inhibit tumor growth. In addition, based on the characteristics of driver gene-positive NSCLC, immunotherapy alone may have a risk of hyperprogression, so combination therapy is more conducive to comprehensive control of tumor development. A clinical study called ORIENT-12 compared the efficacy and safety of sintilimab (ICI) combined with platinum-based drugs and gemcitabine (GP) versus GP alone in patients with locally advanced or metastatic LUSC. The results showed that after a median follow-up of 12.9 months, the PFS of the sintilimab combined with the GP group was significantly better than that of the placebo combined with the GP group, and the incidence of treatment-related adverse events leading to death in the two groups was 4.5% and 6.7%, respectively (26). In this study, the use of sintilimab combined with chemotherapy also achieved favorable immunotherapy outcomes in LUSC patients. This may be due to the high TMB increasing the number of tumor-specific antigens on the surface of tumor cells. These antigens can be recognized by the patient’s immune system as “foreign” substances, thereby stimulating immune cells to attack tumor cells. Additionally, antigens released by tumor cells with high TMB can more effectively activate T cells, leading to a stronger immune response against tumor cells (27). The characteristics of immune-inflammatory liver metastases include a large number of immune cell infiltrations. High levels of *PD-L1* and *CD8* ^+^ infiltrations in the tumor area and stroma are typical features of positive indicators in liver metastases. This suggests that liver metastases may be influenced by T cell immune surveillance and play an important role in the immune-inflammatory microenvironment (28). M1-type macrophages (*CD68* ^+^ *CD163* ^-^) are typically associated with anti-tumor immune responses, while M2-type macrophages (*CD68* ^+^ *CD163* ^+^) are more likely to promote tumor growth and metastasis (29). In this study, both M1 and M2-type macrophages showed good infiltration in the tumor area, suggesting that the immune-inflammatory microenvironment of liver metastases may involve complex interactions between different macrophage subtypes. The presence of very few exhausted T cells may also impact the immune response to immunotherapy in patients with positive indicators of liver metastases, warranting further investigation (30). Therefore, the immune-inflammatory microenvironment is often considered as a predictive factor for a patient’s sensitivity to immunotherapy (31). In an immune-inflammatory microenvironment, immunotherapy may be more likely to stimulate immune cells to attack tumor cells, leading to better treatment outcomes (32).

Related studies have discussed the use of ctDNA as a biomarker in patients with advanced cancer undergoing immunotherapy. One study collected 316 plasma samples from 94 patients, including baseline and sampling every three cycles, and analyzed them using specific ctDNA detection methods. The results showed that the concentration of ctDNA at baseline was correlated with PFS, overall survival, clinical response, and clinical benefit; and this correlation became more significant as treatment progressed (33). Another study analyzed ctDNA samples from 978 patients with 16 types of advanced tumors before treatment and during treatment (171 samples). The results indicated that pretreatment ctDNA levels appeared to have prognostic value in a broad analysis of immune checkpoint blockade, while dynamic changes in ctDNA during treatment could predict treatment benefit (34). In this study, it was found that the level of ctDNA in the blood plasma significantly decreased after treatment compared to before treatment, which was consistent with the radiological evaluation, further supporting the effectiveness of the treatment.

## 4 Conclusion

In conclusion, we have presented a case report of stage IV (cT3N3M1c) LUSC patient who harbored both a rare *EGFR* p.G719C mutation and high expression of *PD-L1* (TPS=99%). Through the combined approach of immunotherapy and chemotherapy, we observed favorable therapeutic outcomes, with evident tumor reduction. This case provides valuable evidence for the feasibility and efficacy of immunotherapy combined with chemotherapy in managing LUSC with a rare *EGFR* mutation and high *PD-L1* expression. Such efforts will contribute to a more comprehensive evaluation of this treatment strategy’s effectiveness and offer more efficient therapeutic choices for lung cancer patients.

## Data availability statement

The original contributions presented in the study are included in the article/supplementary material. Further inquiries can be directed to the corresponding author.

## Ethics statement

The studies involving humans were approved by Institutional Ethics Committee of the Zhongshan Hospital. The studies were conducted in accordance with the local legislation and institutional requirements. The participants provided their written informed consent to participate in this study. Written informed consent was obtained from the individual(s) for the publication of any potentially identifiable images or data included in this article.

## Author contributions

Z-fZ: Formal analysis, Writing – original draft, Writing – review & editing. X-xB: Data curation, Software, Writing – review & editing. H-yS: Data curation, Formal analysis, Investigation, Writing – review & editing. X-xG: Data curation, Formal analysis, Funding acquisition, Investigation, Resources, Writing – review & editing.
